# Ceramide in Coronary Artery Disease: Troublesome or Helpful Future Tools in the Assessment of Risk Prediction and Therapy Effectiveness?

**DOI:** 10.3390/metabo15030168

**Published:** 2025-03-01

**Authors:** Melania Gaggini, Adrian Florentin Suman, Cristina Vassalle

**Affiliations:** 1 Institute of Clinical Physiology, National Research Council, Via G. Moruzzi 1, 56124 Pisa, Italy; melania.gaggini@cnr.it (M.G.); adriansumanflorentin@gmail.com (A.F.S.); 2 Fondazione CNR-Regione Toscana G Monasterio, Via G. Moruzzi 1, 56124 Pisa, Italy

**Keywords:** atherosclerosis, acute myocardial infarction, acute coronary syndrome, ceramides, sphingolipids, prognosis, risk, therapy

## Abstract

Lipids are a complex entity of different molecules, among which ceramides (Cers), ubiquitous sphingolipids with remarkable biological activity, can represent a potential additive biomarker that can be used to better understand the underlying mechanisms which drive the onset and development of atherosclerotic damage and plaque vulnerability and facilitate coronary disease management, as possible risk/prognostic biomarkers and targets for therapeutic intervention. Accordingly, this review aims to discuss the available results on the role Cersplay in contributing to atherosclerosis development and acute coronary event precipitation, their impact on complications and adverse prognosis, as well as the impact of treatment options in modulating Cerlevels.

## 1. Introduction

Low-density lipoprotein cholesterol (LDL-C) is a key well-known causal determinant in the onset and development of atherosclerotic damage [1]. Because of this importance, lipid-lowering therapy, primarily focused on LDL-C levels, is the pillar of the prevention of cardiovascular disease [1]. Nonetheless, in recent years, other lipid biomarkers (e.g., triglyceride (TG)-rich lipoproteins (TGRL) and their remnants, lipoprotein(a) Lp(a)) have been studied as possible indicators not only of atherosclerotic development but also of atherothrombotic acute event precipitation [1]. In fact, lipids are a multifaceted whole of different components; among these molecules, Cers have received great interest as complex ubiquitous sphingolipids with marked biological activity and different effects in many cell pathways (e.g., cell membrane integrity, cellular stress response, inflammation and oxidative stress, apoptosis) [2]. In particular, some data have suggested that Cers may reflect the activity and the vulnerability of the atherosclerotic plaques [3]. Moreover, blood levels of some Cers are associated with different traditional risk factors (e.g., hypertension, type 2 diabetes–T2D) in patients with stable coronary artery disease (CAD) or acute coronary syndrome (ACS) [4,5]. Different studies have reported the strong predictive value of Cers on adverse events in stable and acute CAD [4]. Other findings have found evidence that some interventions, successful in decreasing the risk of adverse events (e.g., diet, aerobic exercise, or pharmacological tools such as statin therapy), may modulate Cerconcentration [6,7,8]. All together these results suggest that Cerlevels can represent a potential additive tool to better understand atherosclerosis pathophysiology and facilitate cardiovascular (CV) management in clinical practice, for example, as risk/prognostic biomarkers and targets for therapeutic intervention to prevent adverse prognosis.

Accordingly, this review aims to discuss the available results on the role of Cers in contributing to atherosclerosis and acute coronary events, their impact on prognosis, as well as the role of treatment options in modulating Cerlevels in this patient population.

## 2. Biological Structure, Effects, and Measurement of Cers

**Figure 1:**
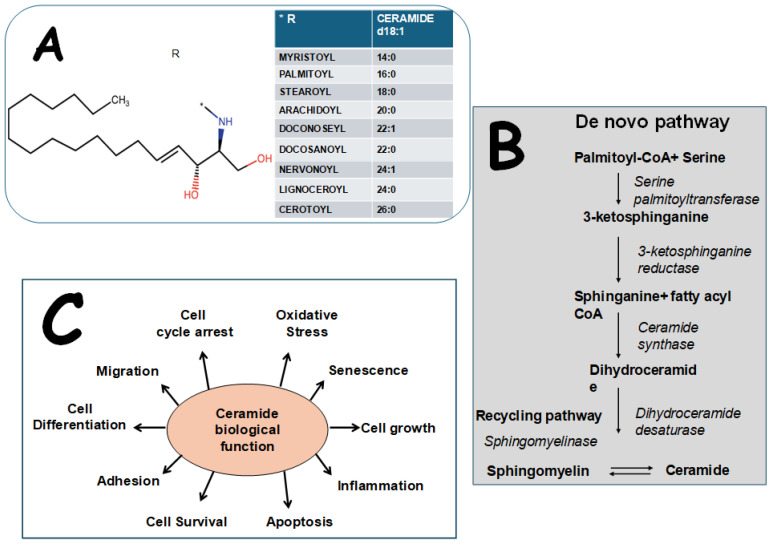
(Panel (**A**)) Ceramide structure. R = fatty acid chain. (Panel (**B**)) Main Cerssynthesis pathways: de novo pathway and recycling pathway. (Panel (**C**)) Main biological function of Cers.

Sphingolipids are an important family of lipids characterized by the presence of sphingosine. Cers consist of a hydrophobic fatty acid chain linked to an amino-containing sphingoid base; the link is obtained via an amide bond between the carbonyl group of the carboxylic acid of the fatty acid chain and the amino group of the base Figure 1 [9,10]. Cers are an integral part of the structure of cell membranes and lipid rafts, having broad biological functions in multiple cell mechanisms. Their biological effects extend from mitochondrial dysfunction and oxidative stress, which can lead to apoptosis, to the modulation of various cellular functions such as inflammation, differentiation, cell growth, cell arrest, senescence, migration, and adhesion [2]. This variety of function arises from the chemical diversity of the family, which sometimes leads to opposite effects on cells. For instance, the balance between long-chain Cers and very long-chain Cers is crucial, as long-chain Cers promote apoptosis, while very long-chain-Cers support cell survival [11]. Importantly, they can activate the NLRP3 inflammasome, leading to cytokine secretion and inflammation by promoting the cleavage of caspase-1 [12] and, additionally, various levels of the MAPK pathways (p38MAPK and JNK), driving further the inflammatory response [13].

Cers accumulation led to Akt (Protein-kinase-B) and HSL (hormone-sensitive-lipase) inhibition, disrupting a key part of the insulin signaling cascade and a key enzyme responsible for lipid mobilization. The resulting inhibitions promote excessive lipid storage within adipose and non-adipose tissues, contributing to metabolic imbalances and the pathogenesis of insulin resistance (IR) and related disorders [14].

Cers are responsible for the uncoupling of the nitric oxide (NO) signaling pathways that lead to platelet activation and endothelial dysfunction, implicated in the progression of atherosclerosis and associated with the development of multiple CV risk factors [4]. These processes highlight the importance of Cers in normal cellular functions and in main chronic degenerative disease pathophysiology, including cardiovascular diseases, T2D, cancer, neurodegeneration, and metabolic disorders [6].

Cersare synthesized through several pathways:

De novo synthesis: this process occurs in the endoplasmic reticulum, starting with the condensation of palmitoyl-CoA and serine by serine-palmitoyl transferase, forming 3-keto-dihydrosphingosine. This intermediate is reduced to sphinganine by 3-keto-dihydrosphingosine reductase and subsequently converted into dihydroceramide through Cersynthase.

The sphingomyelinase/recycling pathway: in the cell membrane, sphingomyelinase enzymes hydrolyze sphingomyelin, resulting in the production of Cerand phosphocholine.

Minor pathways: derived from recycled molecules from the endo/lysosomal pathway, where sphingomyelin is broken down into sphingosine, and then converted intoCer. Furthermore, there are other minor sources that produce Cerfrom glucosylceramide (by glucosylceramidase), galactosylceramide (by galactocerebrosidase), and ceramide 1-phosphate (by ceramide 1-phosphatase), mainly from nervous tissue [10,15] (Figure 1).

The primary biological matrices utilized for Ceranalysis include blood, tissue, cultured cells, and urine. The evaluation of the Cers can be performed by various techniques, from enzymatic diacylglycerol (DAG) kinase assays to liquid–gas and thin layer chromatography coupled with mass spectrometry due to the high specificity and sensitivity. The most used are LC–MS, LC–MS/MS, LC-ESI-MS/MS, gas–liquid chromatography, or fast liquid chromatography quadrupole time-of-flight mass spectrometry [16].

**Figure 2:**
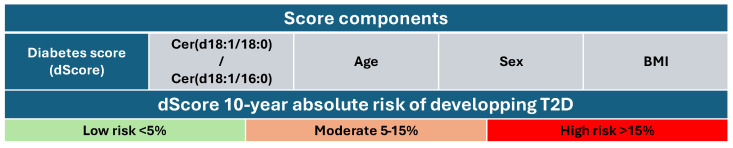
The diabetes score (dScore) incorporates the Cerratio with the addition of other important risk factors (age, sex, and body mass index—BMI), giving the risk of developing T2D in the next 10 years. (scale 0–100). Each subject is assigned to one of the following risk categories for developing T2D in the next 10 years: low risk is defined as <5%, moderate risk 5–15%, and high risk more than 15% probability.

**Figure 3:**
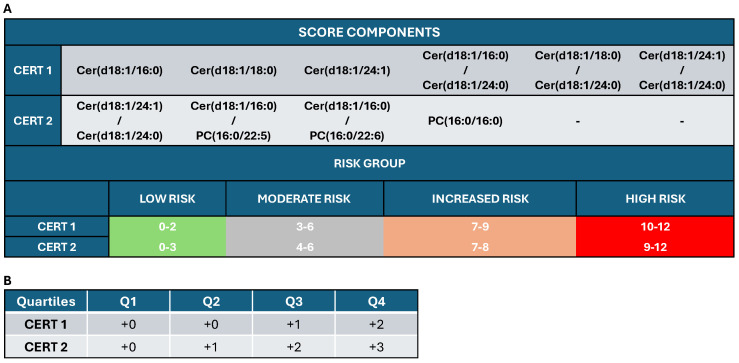
Panel (**A**) The CERT1 score consists of six components: three individual Cersand three Cer/Cer ratios. Subjects are assigned a 13-level score (0–12) and, more generally, to 4 risk categories: 1 = low risk, 2 = moderate risk, 3 = increased risk, and 4 = high risk. CERT2 score consists of four components: one Cer/Cer ratio, two Cer/phosphatidylcholines (PC) ratios, and a single PC; also, in this case, each patient is assigned a 0–12 score and 1 of 4 risk categories (from low to high risk). Panel (**B**) Which quartile the score for a patient belongs to is then determined. For CERT1, only quartiles 3 and 4 give risk points, while for CERT2, the second quartile yields a risk point.

The evaluation of the Cers, combined with each other or with other biomarkers as scores, is continually explored, enhancing our understanding of their role in disease mechanisms. T2D and CV disease (CVD) are characterized by alterations of Cers that cause a deficit in insulin regulation and the activation of inflammation, which contribute to insulin insensibility and cardiovascular damage [17,18]. The risk of developing T2D can be predicted by the diabetes score, which uses a Cerratio ([Cer(d18:1/18:0)/Cer(d18:1/16:0)] combined with age, sex, and body mass index (BMI), giving a moderate (<5%) or high (5–15%) risk for the patient to develop T2D in the coming 10 years [19,20]) **(Figure 2**). A CERT1 score is calculated using three Cers (Cer(d18:1/16:0), Cer(d18:1/18:0), Cer(d18:1/24:1)) and their ratios to Cer(18:1/24:0) **(Figure 3**). This score stratifies the patient in four different groups, from 1 to 4, that are proportional to the risk of CVD [20] (Figure 3). CERT2 is quite similar to CERT1 but it also considers two phosphatidylcholine (PC 16.0/16.0 and PC 16:0/22:5) and refers to the prediction of cardiovascular death prediction, inflammation myocardial necrosis, and also myocardial and renal dysfunction and dyslipidemia [21] (Figure 3).

## 3. Cersand Cardiometabolic Risk and Disease

### 3.1. CV Risk Factors

Interestingly, distinct lipidomic profiles have recently been associated with obesity, dyslipidemia, IR, and T2D not only in adults but also in children, providing new potential effective tools to improve therapeutic options for the management of these conditions [22,23]. Cerlevels correlate with dysglycemia progression (fasting glucose at 5-year follow-up) in the general population without cardiovascular disease, suggesting that they may serve as biomarkers of the onset and progression of clinically significant cardiometabolic conditions [24]. In the T2D clinical setting, Cerswere associated not only with T2D incidence, but were also correlated with main T2D biomarkers, including fasting glucose and insulin and HOMA-IR, as well as with contradictive signs of insulin sensitivity [23,25,26].

Moreover, other cardiovascular risk factors and adverse CV features have been linked to Cerabnormalities, including vascular inflammation, oxidative stress, and the main apoptosis signaling pathways by which Cersexert their effects on cardiometabolic risk and disease [2,5]. Accordingly, experimental and human studies evidenced positive relationships between Cersand blood pressure or vasoconstriction, whereas antihypertensive therapy is often associated with a reduction in Cer levels [27,28].

### 3.2. Atherosclerosis

Endothelial dysfunction is the first feature of atherosclerosis; some results indicate that Cersalso affect blood flow-induced dilation (FID) and NO bioavailability, whereas targeting Cerexcess (e.g., myriocin) restores FID and NO production, protecting endothelial function in human and experimental studies [29,30,31]. The relationship between Cers (especially Cer(d18:1/16:0), Cer(d18:1/18:0), and Cer(d18:1/24:1)) and CVD has been reported in many different case–control and cohort trials [20]. Accordingly, alterations in Cershave been proven in patients with atherosclerosis and CAD [32,33]. Interestingly, specific Cerspecies have shown different patterns in patients with stable or unstable angina and acute myocardial infarction (AMI) and in subjects with different severity levels of coronary stenosis [34]. In fact, acute coronary syndrome (ACS) encompasses various types of myocardial ischemia, such as ST-elevation myocardial infarction (STEMI), non-STEMI, and unstable angina pectoris, and many studies suggest that a dysregulated metabolism of several lipid molecules play significant roles in the formation, rupture, and subsequent development of acute events [35,36]. The evaluation of novel lipid biomarkers, derived from patients’ metabolomic profiling, could thus provide prognostic information, thereby improving risk-stratified patient management in this clinical field [37].

Many studies have discovered an association between CerS and multiple atherosclerotic processes [38,39], since Cers favor the infiltration of oxidized low-density lipoprotein cholesterol (LDL-C) into vascular walls, the expansion of the lipid-rich core, monocyte adhesion, and atherosclerotic plaque formation [40]. Laaksonen et al. have found that certain plasma Cers, such as Cer18:1/Cer16:0; Cer18:1/Cer18:0, and Cer18:1/Cer24:1, and Cer ratios (Cer18:1/16:0)/(Cer18:124:0) Cer18:0/Cer24:0 and Cer24:1/24:0 have been found as independent predictive values for CV events [41]. In 216 patients who were admitted and underwent coronary angiography for suspected CAD, parameters including baseline serum routine lipid markers, inflammatory markers, and Cers have been evaluated. Plasma levels of Cer(d18:1/24:0), Cer(d18:1/22:0), Cer(d18:1/18:0), and Cer(d18:1/20:0) were significantly elevated in ACS patients. The multivariate logistic regression showed that when incorporating the traditional risk factors, Cer(d18:1/16:0), and IL-6, the AUC was 0.827 compared to the AUCs of the models individually considering the traditional risk factors, Cer(d18:1/16:0), or IL-6, which were 0.782, 0.785, and 0.722, respectively. These results can be important because they demonstrate that the simultaneous use of traditional risk factors, IL-6, and Cer(d18:1/16:0) can improve the diagnostic accuracy of ACS [42].

### 3.3. CAD Severity and Plaque Characteristics

In a post hoc analysis of the CorLipid trial, novel biomarkers and metabolomics-based prediction models were evaluated to predict clinical outcomes and assess the complexity of CAD (SYNTAX score). In patients who underwent emergency or elective coronary angiography due to ACS (n = 316), a Cerratio of Cer24:1/C24:0, an acylcarnitine ratio of C4/C18:2, and peripheral artery disease were independent predictors of higher CAD complexity [43]. Also, another study based on the CorLipid trial evaluated the severity of CAD by the use of a machine learning algorithm. In patients grouped into obstructive CAD (SS > 0) and non-obstructive CAD (SS = 0), Cer(18:1/18:0) levels were significantly lower in the SS = 0 group compared to the other group. Patients with stable angina had a significantly lower concentration of Cer(18:1/16:0) and Cer(18:1/18:0) compared to patients with NSTEMI and STEMI. Cer(18:1/24:0) and Cer(18:1/24:1) were substantially higher in STEMI patients compared to patients with unstable and stable angina [44].

In 553 patients with suspected or definite ACS or other CADs who underwent angiography, divided into four groups according to the severity of coronary artery stenosis, Cers(d18:1/16:0), (d18:1/18:0), (d18:1/24:1), and (d18:1/24:0) were carried out by liquid chromatography–tandem mass spectrometry and the ratios of Cer(d18:1/16:0) to Cer(d18:1/18:0) and Cer(d18:1/24:1) to Cer(18:1/24:0) were calculated. The results showed higher levels of Cer(d18:1/16:0) and Cer(d18:1/24:0) and a higher ratio of Cer(d18:1/24:1) to Cer(d18:1/24:0) observed in AMI patients compared to unstable angina patients; moreover, the ratio of Cer(d18:1/24:1) to Cer(d18:1/24:0) was significantly increased in subjects with stenosis > 50% compared to those with stenosis < 50% [34].

In subjects with AMI, Cer(d18:1/16:0), Cer(d18:1/18:0), and Cer(d18:1/24:1) levels and their ratios to Cer(d18:1/24:0) were evaluated, demonstrating for the first time a correlation of Cers with multivessel disease (an index of disease severity), ejection fraction—EF (index of left ventricular contractility), wall motion score index—WMSI (an index of wall motion), and brain natriuretic peptide—BNP (index of wall strain and dysfunction) in an AMI cohort, evidencing a close link between Cers and left ventricular dysfunction and heart failure (HF). In particular, all Cers considered and their ratios were associated with BNP; this, together with the finding that Cer(d18:1/18:0)/Cer(d18:1/24:0) and Cer(d18:1/24:1)/Cer(d18:1/24:0) were associated with elevated troponin (hs-TnT), suggests the possible role of Cers in both myocardial necrosis and dysfunction following AMI [5].

Importantly, circulating levels of Cers were related with morphology, heterogeneity, the instability of the plaque, and the triggering of the acute event, supporting their role as proatherogenic determinants, as well as their potential to represent biomarkers of plaque vulnerability. In the ATHEROREMO-IVUS study, some Cerspecies and ratios, in particular Cer(d18:1/16:0), were associated with the fraction of necrotic core tissue and lipid core burden and with 1-year major adverse cardiac events (MACEs: all-cause mortality, ACS, and coronary revascularization) in acute and stable CAD patients [36]. Circulating levels of Cer(d18:1/16:0), Cer(d18:1/18:0), Cer(d18:1/24:1), and Cer(d18:1/24:0) were found to be higher in STEMI patients compared to normal donors and stable patients and were associated with a higher atherosclerotic burden and adverse prognosis in patients with STEMI [45]. Accordingly, plasma Cerlevels (Cer(d18:1/16:0), Cer(d18:1/18:0), and Cer(d18:1/24:1)) were found to be more significantly associated with plaque rupture than plaque erosion in STEMI patients [45]. At present, the visualization of Cers in human coronary plaques can be performed (e.g., by optical coherence tomography and fluorescent angioscopy) and potentially used to characterize the plaque as a biomarker of vulnerability [3,46].

### 3.4. Outcome

Cershave also shown independent predictive value for adverse cardiovascular outcomes. MACEs (defined as major adverse cardiac and cerebrovascular events 1 year after hospitalization) were investigated, and the prognostic association of individual Cerspecies with MACEs was modeled using multiple logistic regression models. After adjusting concentrations of targeted Cersand other risk factors, the study showed that pre-existing infarction was associated with MACEs, whereas no Cershowed an independent association with MACEs [34]. Another study identified a 12-Cer prognostic panel in paired aortic tissue–plasma and paired myocardial tissue–plasma samples associated with adverse prognosis in AMI, suggesting ischemic myocardium as a possible source of this Cersignature [47].

In patients with ACS, high-performance liquid chromatography with tandem mass spectrometry (LC/MS) was used to measure the plasma levels of 11 Cers (Cer16–Cer26), with the primary outcome being all-cause mortality and the secondary outcome being cardiac mortality during the one-year follow-up. The results indicated that five Cers (Cer 18:1/16:0, Cer18:1/18:0, Cer18:1/20:0, Cer18:1/C24:1, and Cer18:1/24:2) and their ratios to Cer(d18:1/24:0) were independently related with the risk of all-cause death and cardiac death, indicating that these five Cerscould identify the high risk of mortality beyond traditional assessment tools in patients with ACS; in fact, the ROC-AUC for all-cause mortality was greater than that of the GRACE score alone (which estimates the risk of death or death/AMI in ACS patients) [48]. In another study conducted in 581 patients who underwent diagnostic coronary angiography or percutaneous coronary intervention for stable angina pectoris (SAP) or acute ACS, the relationship between three Cerratios with the occurrence of MACEs (all-cause mortality, nonfatal ACS, or unplanned coronary revascularization) during a median follow-up of 4.7 years was investigated. The main finding of this study was that Cer(d18:1/16:0) and Cer(d18:1/24:1) at higher concentrations were significantly associated with MACEs after multivariable adjustment for cardiac risk factors, clinical presentation, and statin use at baseline. Moreover, the concentrations of Cer(d18:1/16:0), Cer(d18:1/20:0), and Cer(d18:1/24:1) and all Cerratios were associated with the secondary endpoint (composite of all-cause mortality or nonfatal ACS), suggesting these lipid species as predictors of long-term clinical outcomes in CAD patients [49].

Interestingly, Lozhkina et al. studied Cers as potential new predictors of the severity of acute coronary events in conjunction with SARS-CoV-2 infection, dividing the patients into two groups: the favorable outcome (recovery) group and the in-hospital fatal outcome group. The results showed that the Cerplasma concentration was significantly lower in the fatal outcome subgroup than in the survivor subgroup. The non-structural SARS-CoV-2 proteins could activate the metabolic pathways involved in apoptosis and inflammation that lead to the depletion of the precursors of these metabolites in the terminal condition, and for this reason, the determination of the Cerconcentration in patients with COVID-19 infection and acute coronary events may help to assess the prognosis of these patients and manage their risks in this particular clinical setting [50].

Designing a metabolic risk stratification for ACS remains a challenging task. ACS patients often have metabolic syndrome (MS), contributing to an increased cardiovascular death rate and long-term adverse events. Dysregulation in Cer metabolism is commonly observed in subjects with metabolic disorders [51,52]. In T2D, the utility of an 11-Cer panel in diagnosing microvascular disease was assessed in 309 patients (healthy controls, noAMI-T2D, and AMI-T2D patients). ROC-AUC values using the 11-Cer panel showed significant improvements in the outcome prediction, suggesting Cersas potential predictors for microvascular disease phenotypes in T2D [53]. Recently, four Cers(Cer(d18:1/16:0), Cer(d18:1/18:0), T2D and1/24:0), and Cer(d18:1/24:1)) and their ratios were evaluated to assess the best predictors of incident T2D in the FINRISK 2002 population; the results evidenced that the Cer(d18:1/18:0)/Cer(d18:1/16:0) ratio represents an independent predictive biomarker for incident T2D and may be changed by lifestyle intervention [19]. In this context, the plasma Cerconcentration was measured in 695 ACS patients, 286 of whom were diagnosed with metabolic syndrome—MS (ACS-MS) and 409 without MS (ACS- noMS), who served as the control group. Compared with the ACS-noMS group, the levels of Cer18:/16:0, Cer18:1/18:0, Cer18:1/20:0, Cer18:1/22:0, and Cer18:1/24:1 in the ACS-MS group were higher, with Cer18:1/18:0 showing a significant sensitivity to minor changes in MS risk status in patients and thus emerging as a promising biomarker for early MS detection and risk stratification [54].

### 3.5. Vitamin D

In subjects with AMI, the link between serum 25(OH)D and Cers was studied by us, since vitamin D can affect the cardiovascular system, specifically acting on cardiac fibrosis, cardiomyocyte proliferation, immune responses, and inflammatory signaling [55]. The data obtained from this study showed that Cer(d18:1/16:0) and Cer(d18:1/18:0) were negatively related with 25(OH)D, whereas AMI patients with adequate 25(OH)D levels (≥30 ng/mL) showed reduced values of Cer(d18:1/16:0) and Cer(d18:1/18:0), which were particularly high in patients with severe hypovitaminosis D (<10 ng/mL). T2D/dyslipidemic patients with levels of 25(OH)D (<30 ng/mL) had higher levels of both Cers when compared with the rest of the population. Moreover, 25(OH)D remained an independent determinant for Cer(d18:1/16:0) and Cer(d18:1/18:0) in this group of patients [56].

**Figure 4:**
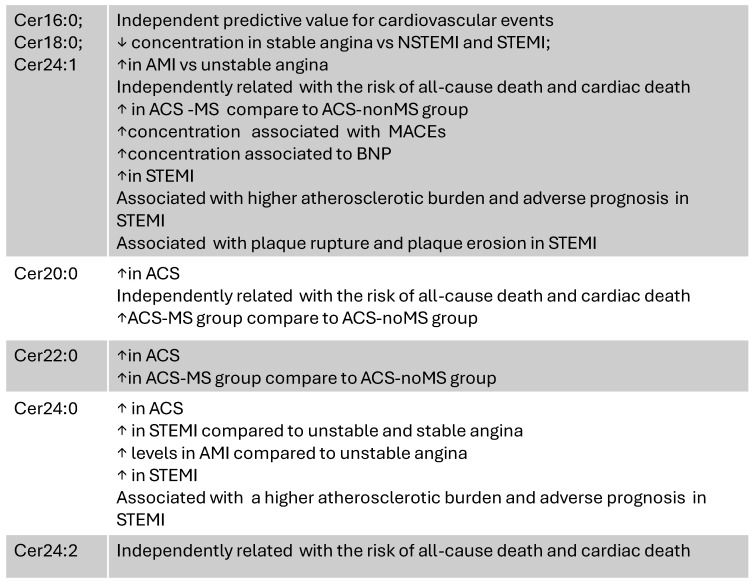
Main data on the role of different Cerspecies in the AMI setting and as predictors of adverse CV events.

Figure 4 reports the main results regarding the role of different Cerspecies in the AMI setting and as predictors of adverse CV events, suggesting their possible utility and potential future application as additive tools in the assessment of disease risk and prognosis in the clinical practice.

## 4. CerModulation

One key promising Cerdevelopment is their use as possible biomarkers to modulate and support conventional treatment in CAD patients. To date, available results in the general population evidence that Cer concentration may be modulated by different strategies, such as caloric restriction, intermittent fasting, diet, and aerobic exercise, as well as by some drugs commonly used in the cardiovascular setting (e.g., statins).

### 4.1. Dietary Effects

Experimental data suggest that the ketogenic diet as well as caloric restrictions are able to reduce Cers in the liver [57,58]. Nonetheless, these changes can be tissue-specific, as caloric restriction did not seem to influence the total Cercontent in myocardial mice or in rat muscle [59,60]. It has also been observed that dietary saturated fatty acid (SFA) promotes liver fat and increases in blood Cers, whereas polyunsaturated fatty acid prevents these events in overweight individuals during excess energy intake and weight gain; interestingly, the adverse effects of SFA were reversed by caloric restriction [61]. Other data showed that intermittent fasting prevents Ceraccumulation in the right ventricle of an experimental model of pulmonary arterial hypertension [62]. Moreover, in subjects with obesity, intermittent fasting has been found to be associated with improvements in plasma sphingosine, sphinganine, sphingomyelin, and dihydrosphingomyelin lipid species, together with the traditional lipid biomarkers and inflammatory parameters [63].

Another study showed that dietary intervention (low-energy diet) significantly modifies levels of Cers and also diacylglycerols, lysophospholipids, and ether-linked phosphatidylethanolamine in subjects with overweight-associated prediabetes, with many lipid species being closely associated with clinical glycemic biomarkers [64]. We also confirmed this association in AMI patients, where some specific Cerspecies were modulated by the presence of T2D [23]. Concerning dietary regimens, one study compared the healthy Nordic diet (including whole grains, fruits, vegetables, berries, vegetable oils and margarines, fish, low-fat milk products, and low-fat meat) against the Nordic diet (characterized by low-fiber cereal products, dairy fat-based spreads, regular-fat milk products, and a limited amount of fruits, vegetables, and berries); the results showed that the healthy Nordic diet may favorably modulate the lipidomic profile, reducing levels of IR-inducing Cers as well as enhancing antioxidative plasmalogens [65]. Interestingly, in vitro data showed that an oversupply of free fatty acids (FFAs), especially long-chain saturated FFAs, promoted Ceraccumulation, whereas unsaturated FFAs (arachidonic acid) are able to somewhat prevent the increase in Cers inducted by saturated FFAs (palmitic acid) [66,67]. Accordingly, an increased intake of SFAs also induced an increase in deleterious Cersand IR in overweight subjects [68]. Instead, the Dietary Approaches to Stop Hypertension (DASH) diet (limits salt, added sugar, and saturated fat intake) modifies levels of Cer, decreasing the total Cerblood levels (Cer22:0 and 24:0 decreased by 27.6% and 10.9% and 24:1 increased by 36.8%) [69].

Several randomized controlled trials (RCTs) highlight how the Mediterranean diet (MedDiet, characterized by a high intake of virgin olive oil, fruit, nuts, vegetables, and cereals; a moderate intake of fish and poultry; a low intake of dairy products, red meat, processed meats, and sweets; and wine, oil, and nuts in moderation) may benefit health, acting on several cardiometabolic risk factors, such as body mass index, waist circumference, hypertension, and T2D [70]. Previous studies have indicated that the beneficial effects of the MedDiet on health could be mediated through many different mechanisms, including improvements in lipid profiles and endothelial function and reductions in oxidative stress and inflammatory status [71]. In the PREDIMED trial (230 incident cases of CVD and 787 randomly selected participants at baseline, followed for up to 7.4 years), a significant positive association between the blood Cerconcentration and higher CVD risk was evidenced; this relationship was attenuated in the groups following the MedDiet adoption [72]. Many of the beneficial effects of the MedDiet are mediated by its key components, such as oleic acid (the major component of extra-virgin olive oil—EVOO), and polyphenols contained in EVOO and red wine (e.g., resveratrol—RSV) [71]. Recent data, obtained in healthy young adults, reported that EVOO-enriched chocolate spread induced a better sphingolipids and glucose profile, decreasing plasma CerC16:0, the CerC16:0/Cer C22:0–CerC24:0 ratio, and sphingomyelin C18:0 when compared to palm oil-enriched chocolate spread consumption [73].

RSV is particularly contained in red wine; recent studies have demonstrated that some RSV beneficial effects on cardiometabolic risk and disease are mediated by changes in sphingolipids [7]. Interestingly, although much knowledge is needed in order to better understand the complexity of RSV effects and different mechanisms involved in the modulation of sphingolipids (dose-effects, cell tissue-specificity, etc.), this relationship has been more studied in the cancer field, where RSV was able to modulate apoptosis and proliferation by acting on the Cerpathway [7,74,75,76].

Recent studies have deepened the relationship between gut microbiome composition and Cers. In the Helius study, the gut microbiome was associated with sphingolipid levels (mainly the relationship with higher levels of Cers and lower levels of more complex sphingolipids), which in turn may affect the onset of T2D [77]. In this context, dietary intervention (omega-3 and fiber supplementation) decreased plasma Cers (d18:1/16:0, d18:0/24:0, and d18:1/24:1), which were associated with changes in the gut microbiome (decrease in Colinsella and increases in *Bifidobacteriuim*, *Coprococcus 3,* and short-chain fatty acids) [78].

There is also an interesting reciprocal influence between vitamin D (mainly produced through sun exposure, but also contained in some food, as some fish, eggs, mushrooms, or fortified milk) and sphingolipids, evidenced both in the cardiometabolic and nervous system [79,80]. As above reported, we recently showed a significant inverse association between 25(OH)D blood levels and both Cer(d18:1/16:0) and Cer(d18:1/18:0) in AMI patients, with the highest levels of the two Cers observed in AMI patients with severe hypovitaminosis D (<10 ng/mL) [56].

### 4.2. Exercise

Another recognized determinant of sphingolipids is aerobic exercise; in the Framingham Heart cohort, the associations of dietary indices and quantitative cardiorespiratory fitness (CRF) were measured in a large population, including 2380 subjects [81]. C16:0 Cerwas associated with lower CRF and poorer dietary quality [82]. A recent systematic review focused on the association between CRF and metabolites measured in human tissues and body fluids; the results, based on 22 studies, evidenced an inverse association between CRF and Cerlevels [81]. In another study, sex- and age-independent relationships were evidenced for higher C24:0 and C24:0/C16:0 ratios with higher maximal oxygen consumption, oxygen consumption at the anaerobic threshold, and the association between C24:0 and C16:0 with maximum workload (Wattmax kg-1). Differences related to aging and sex were found, with an inverse association found between Wattmax kg-1 with C22:0 in women < 54 years, whereas a positive association was observed in men ≥ 54 years. Moreover, a direct association was found between C24:0 and Wattmax kg-1, but not for women < 54 years, where no association was observed [83]. Nonetheless, when the effect of a 12 week very low-carbohydrate high-fat (VLCHF) diet and high-intensity interval training (HIIT defined as periods of intense activity separated by low-intensity breaks) on lipidomic and metabolomic profiles in overweight and obesity individuals was investigated, the dietary regimen considerably affected the lipid profile while the HIIT effect was much less significant; in addition, no synergistic effect of the VLCHF diet and HIIT on the lipidomic and metabolomic profiles was found [84]. There are also data in patients with CAD, where an improvement in VO2peak was associated with a decrease in CersC16:0 [85].

In view of the available evidence, CRF seems to be associated with the circulating lipidome composition; however, discrepancies between studies highlight the complexity of the relationship between exercise and Cers, which clearly also depends on the training, type, intensity, and duration of the physical activity performed. Moreover, the causality of these associations remains to be clearly demonstrated, as do the specific species involved, considering that studies are heterogeneous in terms of participants’ characteristics and the analytical and statistical approaches [81].

In a previous study, we observed that multiple Cerspecies decreased trained half-marathoners after a run, these changes not being associated with variations in inflammatory parameters (transient increase in IL-6 and CX3CL1 after the race), suggesting their involvement in beneficial training effects [86]. Instead, when the effect of acute exercise (1.5 h at 50% Vo2 max) and recovery on serum Cer was evaluated in sedentary obese individuals, endurance-trained athletes, and T2D patients, the results showed an increase in blood Cer during exercise and recovery in all groups combined, although most of these results can be explained by the presence of T2D; in particular, C18:0 and C24:1 remained significantly increased in T2D compared with the other two groups during exercise and recovery [87].

### 4.3. Inactivity

Conversely, experimental data showed that 14 days of inactivity can elevate Cers, worsening glucose tolerance, whereas 5 days of bed rest in elderly subjects resulted in whole body glucose dysregulation, impaired skeletal muscle insulin signaling, and the upregulation of muscle IL-6 and Cerbiosynthesis signaling; specifically, MyD88 (an adaptor for inflammatory signaling pathways downstream of members of the Toll-like receptor and interleukin-1 receptor families) signaling increased the expression of SPT2 (the enzyme controlling Cerproduction), demonstrating that it is a key factor in regulating inflammation, Cerbiosynthesis, and impaired insulin signaling in muscles following physical inactivity in both rodent and human models [88]. When the effect of 5 days of bed rest on circulating Cers was evaluated in healthy younger and older adults, an increase in the CERT1 score and Cerratios (C16:0/C24:0, C18:0/C24:0, and C24:1/C24:0) in older but not younger adults was observed; moreover, blood adiponectin (a biomarker of cardiometabolic function) decreased following 5 days of bed rest in both young and older subjects, and these levels were inversely correlated to C16:0/C24:0 and C20:0/C24:0. In this study, the Cerchanges in older adults may reflect the increased CVD risk, while the association between adiponectin with Cerratios may identify this relationship as a determinant mechanism contributing to bed rest-induced deteriorations of CV function [89].

### 4.4. Drugs

Statins, widely used and well recognized as being beneficial in the cardiometabolic field, are also able to modulate plasma Cer levels. In the Ludwigshafen Risk and Cardiovascular Health (LURIC) study, simvastatin (40 mg for two weeks) administration significantly decreases plasma Cers in CAD patients [90]. Taking rosuvastatin (10 or 40 mg/d) for 5 weeks significantly reduced blood sphingolipids and phospholipids (Cer: −33 and −37%, respectively) in men with metabolic syndrome [91]. Moreover, PCSK9 inhibition (RG7652) in CAD patients significantly modifies the lipid composition of plasma and lipoprotein particles, with significant reductions in long- and very long-chain Cers [92]. In addition to lipid-lowering drugs, metformin and pioglitazone can reduce the Cerconcentration, these changes being associated with beneficial clinical responses, as those observed in IR and adiponectin levels were [20]. Moreover, liraglutide (a GLP-1 receptor agonist) decreased Cer levels, phospholipids, and triglycerides, which are all related to a higher CV risk in patients with T2D [93].

We observed a significant reduction in Cer(d18:1/22:0), Cer(d18:1/23:0), Cer(d18:1/24:0), and Cer(d18:2/22:0)) in AMI patients taking lipid-lowering therapy (mainly statins), while antiplatelet therapy (mainly aspirin) was associated with a reduction in Cer(d18:1/24:0) and Cer(d18:1/25:0); instead, no significant association was found between antihypertensive (diuretics, calcium channel blockers, ACE inhibitors, and beta-blockers) and antidiabetic therapy (insulin or oral hypoglycemic therapy) and Cerlevels [23]. In this context, previous results indicated that aspirin at high doses may reduce the blood sphingosine-1-phosphate (S1P) and sphinganine-1-phosphate (SA1P) concentration in healthy volunteers, which, in this case, might be adverse as S1P is cardio-protective [94].

The downregulation of specific sphingolipid species through the inhibition of key enzymes in the Cerpathways are now under study, e.g., myriocin (an inhibitor of serine palmitoylCoA transferase), SPT (a rate-limiting enzyme of the de novo pathway), fenretinide (an inhibitor of dihydroceramide desaturase 1-DES1), S1P receptor agonists (e.g., FTY720, CYM5442, KRP-203), methotrexate (which targets CERS6), as well as strategies to enhance CDase activation to promote Cerdegradation (e.g., the overexpression of AdipoR1 or AdipoR2 in either the adipocyte or hepatocyte) [95,96,97].

Thus, all together, these determinants, which may possibly affect Cerlevels and composition, may represent highly relevant tools for the development of interventions and therapeutics in the cardiovascular setting, in addition to traditional pharmacological treatment.

### 4.5. CerModulation in AMI and HF

There is very little data on the pharmacological modulation of Cers in humans in the acute cardiovascular field, but some interesting observations are emerging in the experimental studies. The available results suggest that cardiac Cers are high after AMI by increased de novo Cersynthesis, and this event is associated with higher cell death rates in the left ventricle and cardiac dysfunction, whereas counteract de novo Cersynthesis may benefit heart function after AMI, inducing cardioprotection. Accordingly, different findings support the idea that Cersmay drive cardiomyocyte dysfunction at the basis of HF. Cerhas been recognized as a cardiotoxin engaging a cardiomyocyte-specific GPI-anchored lipoprotein lipase to enhance Cers and other lipids and inducing lipotoxic cardiomyopathy; de novo synthesis inhibition by treatment with myriocin or genetically by the heterozygous deletion of LCB1 (a subunit of SPT) preserved systolic function and prolongs life in this lipotoxic HF model [98].

A study focusing in vitro on animals and patients explored this tissue at different levels. Specifically, in patients with advanced HF, lipidomic analysis revealed increased total and very long-chain Cersin myocardium (samples obtained before and after ventricular assist device placement) and serum. In mice, SPT and Cerswere found to be increased following AMI (2 and 10 weeks); in this experimental model, a blockade of SPT with myriocin decreased Ceraccumulation in ischemic cardiomyopathy and reduced C16, C24:1, and C24Cers, as well as improving post AMI ventricular remodeling, fibrosis, and macrophage content. Moreover, the genetic deletion of the *SPTLC2* gene (encoding for SPT) protects cardiac function after AMI. In addition, in vitro studies revealed that changes in Cer synthesis are linked to hypoxia and inflammation [99]. Accordingly, another study performed on mice evidenced that the intraventricular administration of myriocin at reperfusion downregulated Cers, improved heart function and reduced the infarct area and inflammation and oxidative stress levels following myocardial ischemia/reperfusion injury [100]. Interestingly, the activation of cardiomyocyte Krüppel-like factor-5 (a factor which regulates cardiac fatty acid metabolism via the direct activation of peroxisome proliferator-activated receptor) is found in HF patients and mice with ischemic cardiomyopathy, associated with increased Cer biosynthesis; the genetic (deletion of KLF5 gene) or pharmacological (ML264) inhibition of KLF5 in AMI mice prevents Cer accumulation, reduces cardiac remodeling, and improves the ejection fraction [101].

Another recent experimental study evidenced that several genes involved in de novo Cersynthesis were upregulated and that Cer(C16, C20, C20:1, and C24) increased 24 h after AMI in mice. The enhanced activity of acid ceramidase (AC—an enzyme that catalyzes Cerhydrolysis to free fatty acids and sphingosine, which is then phosphorylated by Sphk 1 and 2 to generate S1P; modRNA-treated mice) decreases Cers and inflammation and improves heart function and survival, with reduced scar size when compared to the control mice. Moreover, in vitro experiments evidence that AC overexpression increased cardiomyocyte cell survival after hypoxia [102]. Interestingly, S1P lyase (SPL) activity was shown to be significantly upregulated in the cardiac tissue during ischemia, and the pharmacological inhibition of SPL with tetrahydroxybutylimidazole (THI) mitigated those effects [103]. Accordingly, other experimental data indicate that the pharmacological elevation of bioactive lipid levels (through the administration of the SPL inhibitor tetrahydroxybutylimidazole for 3 days, starting at day 4 after AMI) seems to be beneficial in the early phase after cardiac ischemic injury, likely by facilitating S1P elevation; mice treated with THI showed better recovery of cardiac functional parameters and a reduction in scar size [104]. However, other results indicate that in the post-ischemic heart of mice, the increase in Cers appears to be mediated more by acid sphingomyelinase than by de novo sphingolipid synthesis, but the inhibition of acid sphingomyelinase and the consequent reduced Cer levels do not improve heart function or survival after AMI, opening doubts about the final benefits of targeting acid sphingomyelinase as a treatment for the ischemic heart disease [105].

## 5. Present and Future

Some leading scientific societies (e.g., European Atherosclerosis Society and the European Federation of Clinical Chemistry and Laboratory Medicine) have encouraged the identification of innovative lipid biomarkers beyond standard lipid risk factors (e.g., LDL), which may refine the power of risk assessment in certain patient populations [106].

These additive lipid parameters may include sphingomyelin, phosphatidylcholines, and Cers. In particular, Cers have been shown to improve cardiovascular risk prediction for primary and secondary prevention when traditional lipids (e.g., LDL) can be less effective (e.g., in elderly, T2D patients, high-risk patients who need and benefit from aggressive and timely treatment more, stable CAD patients over treatment with statins) [8,41,107,108]. In fact, many results indicate that the risk conferred by certain individualCers, Cerratios, and scores is independent from conventional risk factors, accounting for the residual risk in different clinical cardiometabolic settings [21,109].

Generally, the circulating Cerspecies most commonly involved in cardiometabolic risk and adverse outcomes are elevated levels of CerC16:0, C18:0, C20:0, and C24:1. Cer-lowering strategies, whether interventional or pharmacological, may modulate sphingolipids, improving cardiometabolic risk and outcomes. This possibility represents an interesting point which will require particular attention in future studies. Interestingly, deepening gene variants associated with Cerlevels (e.g., in the relationships with T2D and CAD), which are emerging, might serve as potential future therapeutic targets for Cermodulation [110,111].

Implementing tests for Cers in the clinical laboratory practice may be possible in the near future following the course of other analytes (e.g., hormones and vitamin D), which are now performed daily at high volumes with acceptable costs [2]. Nonetheless, many issues remain to be faced in future studies, which currently oppose the diffusion of their measurements in routine laboratories and utilization in the clinical practice. Cer measurements require specialized instrumentation (mass spectrometry is not available in all clinical laboratories and needs careful maintenance, technical expertise, and skilled operators), costs are still elevated, and there are no shared Cer reference values (perhaps their definition in terms of gender and age intervals will be useful), while there is also a lack of standardization [2]. The identification of a restricted number of relevant species and/or their ratios in panels/scores (also in combination with other anthropometric, blood, clinical, or instrumental biomarkers) may favor their adoption in routine clinical practice, representing a future research objective, as well as because problems of interpretation by clinicians, caused by result complexity, still persist.

Moreover, finding opportunities to target specific species could avoid domino adverse effects, although the complexity of Cer actions (some species increase, others decrease, and there may be compensation between favorable/harmful effects) and different biological effects (long-chain Cers[C16–18] may be adverse, while very long-chain Cers[C20–26] beneficial), together with the fact that culprit species may likely vary across different clinical populations, make this objective difficult to achieve, requiring great efforts in future work [2].

Other important points to overcome will be the assessment of the safety and effectiveness of Cer biosynthesis inhibitors and the definition of intervention timing for different pathophysiological clinical settings. In this context, it will be interesting to collect further knowledge on the specificity of therapeutic interventions (e.g., statins, PCSK9 inhibitors) and answer the question on how (and whether) interventions can directly modulate the circulating and tissue Cer profiles and if this translates into an improvement of cardiometabolic health. Then, are reductions in Cers a direct effect of these therapies or are they secondary to broader lipid-lowering mechanisms? Clarifying this distinction in the future would improve the mechanistic interpretations of therapeutic strategies.

## Data Availability

No new data were created or analyzed in this study.
